# Effect of Soy Lecithin Supplementation in Beef Cows before Calving on Colostrum Composition and Serum Total Protein and Immunoglobulin G Concentrations in Calves

**DOI:** 10.3390/ani10050765

**Published:** 2020-04-28

**Authors:** Edyta Wojtas, Andrzej Zachwieja, Eliza Piksa, Anna E. Zielak-Steciwko, Antoni Szumny, Bogdan Jarosz

**Affiliations:** 1 Department of Cattle Breeding and Milk Production, Wroclaw University of Environmental and Life Sciences, 50-375 Wroclaw, Poland; 2 Department of Animal Nutrition and Feed Management, Wroclaw University of Environmental and Life Sciences, 50-375 Wroclaw, Poland; 3 Department of Chemistry, Wroclaw University of Environmental and Life Sciences, 50-375 Wroclaw, Poland

**Keywords:** nutrition, soy lecithin, calving, blood, passive transfer

## Abstract

**Simple Summary:**

Until recently, fat supplements were considered merely as a source of energy for cows during transition. However, individual fatty acids included in fat supplements may clearly induce different production and metabolic responses, which in consequence change the nutritional value of bovine colostrum. Therefore, it is necessary to consider the type of fat additive to use in feed ration. This study aimed to determine an effect of soy lecithin supplementation on beef cow’s colostrum composition and selected blood parameters in their calves. Obtained results suggest that soy lecithin addition in cows before calving has a beneficial impact on colostrum composition. This concerns mainly an increase of linoleic acid in colostrum, which may influence IgG activity in calf serum. In turn, higher content of these components in colostrum may contribute to improve calves’ survival rate during the first weeks of their life.

**Abstract:**

The aim of this study was to investigate the impact of soy lecithin supplementation in beef cow’s nutrition on colostrum composition and serum concentrations of immunoglobulin G (IgG) and serum total protein (STP) in calves. Twenty pregnant Charolaise cows were assigned to two groups. In the supplementation group (n = 10) during the last four weeks of pregnancy, soy lecithin was administrated in an amount of 20 g/cow/day. In both groups, basic composition (protein, fat, lactose, dry matter), somatic cell count (SCC), total bacteria count (TBC), IgG concentration, and fatty acids profile were determined in colostrum samples. Moreover, STP and IgG concentration were measured in calves’ blood samples on the 3rd, 7th, 14th, and 21st days of life, mothered by supplementation and control cows. Animals fed with soy lecithin before calving produced colostrum with a higher (*p* = 0.049) level of linoleic acid (C18:2 n-6). In addition, these results showed that soy lecithin supplementation has contributed to an increase (*p* = 0.029) of serum IgG in calves on the 14th day of life. The impact of such change in colostrum on IgG levels on calves serum and their half-life need further analysis.

## 1. Introduction

Fat, which is present in high concentration in bovine colostrum, is a necessary ingredient of diet for newborn calves in their first days of life [1]. Importantly, it is not only a source of energy and a substrate for metabolic processes [2], but also an essential factor protecting calves from pathogenic microorganisms, aside from the commonly described immunoglobulins [3]. Recent studies have shown that unsaturated fatty acids (UFA), including linoleic acid and linolenic acid, play an important role in metabolism regulation and cell membrane functions [4]. Deficiency of these unsaturated fatty acids in calves’ nutrition leads to impaired functions of membranes, demonstrated by symptoms such as rough hair coat, alopecia, dry and scaly skin, dermatitis, excess water loss, and impaired nervous function [5]. Despite lack of information about the demand of calves for specific fatty acids, some scientific reports have shown that the addition of linoleic acid (C18:2) and α-linolenic acid to the feed ration contributes to increased daily gain of body weight and feed intake during the first week of calves’ life [4]. Moreover, in the presence of these fatty acids a greater efficiency of immunoglobulins absorption was observed in newborn calves [6].

The fatty acids profile is characterized by a high variability. Both, in milk and colostrum, there is a predominance of short chain saturated fatty acids which are synthesized de novo in the mammary gland [7]. Basic substrates for their production are acetic acid and β-hydroxybutyric acid which are produced in the rumen as a result of the distribution of roughages. Some quantities of β-hydroxybutyric acid can be also created during ketogenesis. In turn, unsaturated fatty acids are derived from dietary sources and distributed to the mammary gland with blood [8], which indicates the need of using feed additives with a high proportion of these essential fatty acids in cow’s feeding. 

Soy lecithin is a mixture of phospholipids, phosphatidylcholine, phosphatidylethanolamine, and phosphatidylinositol [9]. Because of its structural and composition properties, it is used in animal nutrition as a supplement supporting liver function, milk production, as well as fertility and reproduction [10,11,12]. Furthermore, thanks to its high levels of linoleic acid, soy lecithin can influence colostrum quality and immune system of newborns [13]. Therefore, the aim of the present work was to investigate the impact of soy lecithin supplementation in nutrition of beef cows on colostrum composition and the levels of immunoglobulin G (IgG) and serum total protein (STP) fractions in calves’ serum.

## 2. Materials and Methods 

### 2.1. Farm Conditions and Animals

The present study was conducted on a commercial beef farm. Twenty healthy and pregnant Charolaise cows were included in the analysis. Cows were selected based on the parity (between 2nd and 4th parity), as well as the body weight (600–700 kg) and were divided into two groups: I—control group (n = 10) and II—supplementation group (n = 10). The animals were housed in a free-stall system on deep litter. In both groups, the daily ration contained a meadow hay (*ad libitum*) and the addition of concentrate (1 kg/cow/day). For the last four weeks of pregnancy, animals in the supplementation group received powdered soy lecithin (20 g/cow/day), added to the concentrate. All calving was supervised. Newborn calves were fed with colostrum from the udder, within 1 h after birth, and they remained with their mothers in individual pens until 7th day of life (cow-calf bond creation time). After this time, each cow and her calf were reintroduced to the herd. All experimental procedures were licensed by the 2nd Local Ethical Committee for Experiments on Animals in Wrocław, Poland (No. 67/2015).

### 2.2. Samples Collection and Analysis

Colostrum was collected, to sterile containers, from each cow immediately after calving and prior to calf suckling. Each sample (250 mL) was milked from all four quarters. Further, the colostrum samples were cooled to 4–6 °C and frozen at −20 °C for subsequent examination. Prior to analysis, samples were gradually thawed at 4 °C. Once thawed, the content of dry matter, protein, fat, and lactose were determined using Infrared Milk Analyser 150 (Bentley Instruments, Chaska, MN, USA). Somatic cell count was analyzed with Somacount 150 (Bentley Instruments Inc.) and total bacteria count using Bactocount 70 (Bentley Instruments Inc.). Moreover, colostrum IgG concentration was determined by electrophoresis on polyacrylamide gel in the presence of sodium dodecyl sulfate (SDS-PAGE) as was described previously by Pecka et al. [14]. Briefly, 2% SDS was added to defatted and deprived of salt samples. After incubation, 5% β-mercaptoethanol and 0.5% bromophenol blue were added. Electrophoresis was conducted for seven hours (including 3 h of pre-electrophoresis without samples) at a voltage of 280 V. Obtained gels were dyed with Coomassie Brilliant Blue. For quantitative and qualitative evaluation electrophotograms were analyzed using Bio-Rad 6 software (Bio-Rad Laboratories, Hercules, CA, USA). The fatty acids profile in colostrum samples was evaluated using 7890 gas chromatography with a flame ionization detector (Agilent Technologies, Santa Clara, CA, USA) using a capillary column HP–88 (100 m × 0.25 mm × 250 µm). Isolation of fat colostrum was performed according to the Folch method [15]. All steps were conducted as previously described by Kęsek et al. [16]. 

Blood samples were collected from all calves on the 3rd, 7th, 14th, and 21st days of life, from the external jugular vein (10 mL/sample) using hypodermic disposable needles (1.2 × 40 mm, Supra, TSK Laboratory International, Vancouver, BC, Canada) and polypropylene tubes with coagulation activator (Separmed, FLMedical, Torreglia, Italy). Two hours after collection, samples were centrifuged for 5 min at 3000 *g* for serum, which was frozen at −20 °C for subsequent examination. Analysis of STP in serum samples was performed by colorimetric method using biochemical analyser Pentra 400 (Horiba ABX, Montpellier, France) with Horiba ABX reagents. Prior to measurement, calibration and quality control were run according to manufacturer’s instructions. The serum IgG concentration was determined using Bethyl Bovine IgG ELISA Kit (Bethyl Laboratories, Montgomey, TX, USA) according to producer protocol. The serum samples were diluted 1:1000 and a standard curve was generated for each set of samples. The absorbance was recorded by an EPOCH microplate reader (BioTek, Winooski, VT, USA) at 450 nm wavelengths. Intra- and inter-assay coefficients of variation (CV) were both <10%.

The fatty acid profiles in all used feeds and soy lecithin supplement were examined using 7890A gas chromatograph with flame ionization detector (Agilent Technologies, Santa Clara, CA, USA) as described by Maślak et al. [17]). The fatty acids profile in all used feeds and supplement is presented in Table 1.

### 2.3. Statistical Analysis

Data on colostrum were subjected to one-way analysis of variance (ANOVA) using Statistica 10.0 software (StatSoft Poland, Cracow, Poland). In turn, results on calf serum IgG and STP were analyzed using a repeated-measures ANOVA. Before analysis, normal distribution of all data was confirmed using the Shapiro-Wilk test. Furthermore, data for SCC and TBC were transformed to the natural logarithm (log10). This procedure was recommended by Ali & Shook [18] to increase sensitivity of statistical test. Significant differences between groups were evaluated using Duncan’s test.

## 3. Results and Discussion

### 3.1. Colostrum Composition

The levels of basic components in colostrum (Table 2) were similar to the results obtained by other authors [19,20]. However, statistical analysis did not show any significant differences between groups. Notwithstanding, a tendency of slightly higher IgG concentration (*p* > 0.05) was observed in colostrum from cows supplemented with soy lecithin, rich in linoleic acid (C18:2 n-6) compared to control group, respectively 64.84 ± 5.96 g/L and 60.55 ± 15.41 g/L, although without the statistical significance. These results are consistent with Corino et al. [21], who indicated that prepartum supplementation with C18:2 n-6 in sows increased endogenous conjugated linoleic acid (CLA) production, which may lead to increase IgG concentration in colostrum. It is suggested that greater antibody responses could have been mediated by CLA formed during ruminal biohydrogenation from C18:2 n-6 [22]. Studies conducted on animals and human [23,24,25] demonstrated that two active CLA isomers (*cis*-9, *trans*-11 and *trans*-10, *cis*-12) are involved in production of interleukins (IL-10), which regulate immunoglobulin synthesis.

### 3.2. Fatty Acids Profile in Colostrum

The effects of soy lecithin on saturated fatty acids profile in colostrum are shown in Table 3. Among fatty acids, the highest content in total amount of fat was observed for palmitic acid (C16:0) in both groups, however, these differences were not significant. Other studies [26,27,28] have found also marked concentrations of this fatty acid in cow’s colostrum during the first 24 h of postpartum (within the range 28.7–30.9%). This can be caused by the high proportion of roughages in cow’s diet. Furthermore, besides de novo synthesis, a large proportion of C16:0 derived from blood circulatory or body lipids, which may explain the high contents of C16:0 in the first colostrum [29,30].

Feeding animals with different lipid additives alters the fatty acid profile of tissues, colostrum, and milk [31,32]. The obtained results indicated that colostrum from cows fed with soy lecithin had almost similar overall level of unsaturated fatty acids (25.29%) compared to control group (24.72%) (Table 4). Although Mašek et al. [33], who used in their research fat supplement with high proportion of docosahexanoic acid (DHA) and eicosapentanoic acid (EPA), obtained significantly higher UFA level in supplementation group. Furthermore, in the present study, colostrum of supplementation cows had significantly higher level of caproic acid (C6:0; *p* = 0.005), caprylic acid (C8:0; *p* = 0.010), pentadecanoic acid (C15:0; *p* = 0.033) and stearic acid (C18:0; *p* = 0.021). These results are consistent with observations recorded by Santschi et al. [34] who indicated higher proportions of C6:0, C8:0, and C18:0 in cow’s colostrum supplemented with extruded linseed rich in α-linolenic acid. Generally, C4:0 to C15:0 fatty acids are synthesized de novo from acetate, which is a final product of fibre fermentation [35]. Therefore, cow’s feeding with meadow hay is considered as the main reason of these fatty acids increased in milk. In turn, as demonstrated in many studies [36,37,38], fat supplementation rich in PUFA (linseed, sunflower and fish oils) decrease de novo synthesis of C4:0-C15:0 in the mammary gland, which is in opposite to results obtained in the present study. During the transition period, ruminants normally mobilize extensively triglycerides from their body fat reservers, mainly in the form of C16:0, C18:0, and C18:1 cis-9 [39]. Such high uptake of these fatty acids by mammary gland tissue inhibits de novo synthesis of short chain fatty acids. Moreover, increased level of C18:0 in colostrum from supplementation group is probably due to an increased supply of linoleic acid from soy lecithin in feed. According to Hur et al. [40], linoleic acid released from dietary lipids due to activity of bacterial lipase in ruminant’s digestive tract, undergoes further biohydrogenation. This leads to formation of C18:0 which is very important substrate for oleic acid (C18:1 n-9*c*) synthesis. Moreover, high supply of linoleic acid also can contributed to an increase in the concentration of vaccenic acid (C18:1 n-11t) in the rumen [41], the excess of which is transported to the mammary gland, where it is subsequently converted to CLA (*c*-9, *t*-11) by Δ9-desaturase [42]. In the present study, no significant differences for C18:1 n-9*c* and CLA (*c*-9, *t*-11) were obtained. In case of other UFA, lower concentration of palmitoleic acid (C16:1; *p* = 0.005) was observed in colostrum from supplementation cows. Castro et al. [43] noted a similar trend in animals fed with soybean and linseed oils application in diets. C14:1 and C16:1 fatty acids are synthesized from C14:0 and C16:0 by Δ9-desaturase in the mammary gland. Corl et al. [44] demonstrated that lower content of these fatty acids in milk is equivalent to decrease of Δ9 desaturase activity. Moreover, in the present study, higher concentration of linoleic acid (C18:2 n-6; *p* = 0.049) was detected in cow’s colostrum from supplementation group. Additionally, elaidic acid (C18:1*n*-9t) was only detected in the colostrum of supplementation cows, which may indicate that increased intake of polyunsaturated fatty acids (PUFA) in feed allows the PUFA to pass rumen without biohydrogenation and get to the mammary gland, where they can be added to the colostrum. Proell et al. [45], demonstrated that increased level of C18:1 n-9t in milk is connected to the reduction of bacterial biohydrogenation intensity.

### 3.3. IgG and STP Concentrations in Calves’ Serum

For successful transfer of passive immunity, the IgG level in calves’ serum should be at least 10 g/L between 24 and 48 h of life [46,47]. According to Teixeira et al. [48], a rapid decrease of IgG indicates an earlier critical period in which calves can be more susceptible to infections. In the present study, the mean concentration of IgG was higher and far exceeded the suggested value in both groups (Table 5). In the following days a decrease of IgG level was observed in all calves. Interestingly, a significant decrease (−6.09 g/L; *p* = 0.038) between concentration in 7th and 14th day after birth was observed only in the control group (data not shown). This may indicate IgG half-life shortening. According to Murphy et al. [49], IgG catabolism in calves depends on the level of their passive immunity (measured on 3rd day of life) and origin of immunoglobulins. The Authors have shown that in calves with the highest serum immunoglobulin levels, derived from maternal colostrum, the IgG half-life was the longest and lasted for 28.5 days. Previous studies reported IgG half-life of 20 days in calves [50]. Some authors indicted that IgG half-life in calves serum depends on nutrition quality of mothers before parturition [51,52]. Furthermore, in the present study, calves mothered by control cows had significant (*p* = 0.029) lower concentration of serum IgG on the 14th day of life compared to calves mothered by supplemented cows. Generally, a tendency of slower decrease of IgG concentration in subsequent time-points was observed in calves from supplementation group. Normally, fully active immunity in calves’ body starts about 21st–28th day of life. It may suggest that higher level of linoleic acid in colostrum influences the time of activity of mothered IgG in calves serum or their absorbance from serum by calves. Presumably, the statistically higher level of IgG in day 14th in supplementation group occurs due to switch from colostral to inner IgG as day 14th is the begging of the switch.

As STP concentration is strongly correlated with IgG, the STP measurement can be used to evaluate transfer of passive immunity in calves. In the present study statistical analysis did not show any significant differences in calves’ STP concentration between groups, on day 3rd, 7th, 14th, and 21st after birth (Table 6). On the 3rd day of life, STP concentration in serum of calves mothered by control and supplemented cows was the highest, respectively 66.66 ± 10.61 g/L and 67.39 ± 12.88 g/L. These results are consistent with observations made by Villarroel et al. [53] who reported that at 2nd and 3rd days of calves’ life the STP concentration was the highest. In the present study, a decrease of STP level in calves after 3rd day of life was observed, however no significant differences between the groups were detected. The concentrations on the 3rd day in the present study exceeded the minimum value (50–52 g/L) suggested by other authors [54,55,56,57,58].

## 4. Conclusions

In conclusion, the use of soy lecithin in beef cows’ nutrition before calving contributes to an increase of linoleic acid in colostrum, which may influence slower decrease in calves’ serum IgG concentration during the first weeks of life.

## Figures and Tables

**Table 1:** Fatty acids composition in all used feeds and soy lecithin supplement.

Fatty Acids (g/100 g)	Feeds		
	Meadow Hay	Concentrate	Soy Lecithin
C14:0	0.86	0.34	0.08
C15:0	ND	ND	0.06
C16:0	21.43	10.27	20.46
C17:0	5.29	ND	0.16
C18:0	ND	1.21	4.67
C18:1 n-9c	ND	40.58	9.38
C18:2 n-6	34.48	39.62	56.95
C18:3 n-6	0.21	0.04	8.17
C18:3 n-3	33.84	7.94	0.07
Other	3.89	ND	ND

ND: not detected.

**Table 2:** The basic composition (mean ± SD), somatic cell count, total bacteria count and IgG concentration in colostrum from Charolaise cows assigned to control (n = 10) and supplementation (n = 10; diet supplemented with soy lecithin) group.

Components	Group		*p*-Value
	Control	Supplementation	
	X ± SD	X ± SD	
Dry matter (%)	18.34 ± 1.63	17.02 ± 1.62	0.126
Fat (%)	3.55 ± 0.23	3.74 ± 0.26	0.155
Lactose (%)	3.61 ± 1.30	2.89 ± 1.02	0.238
Protein (%)	10.31 ± 1.01	9.72 ± 1.74	0.421
IgG (g/L)	60.55 ± 15.41	64.84 ± 5.96	0.147
LogSCC	3.11 ± 0.43	3.09 ± 0.49	0.928
LogTBC	2.64 ± 0.95	2.71 ± 0.84	0.863

X: means; ±SD: standard deviation.

**Table 3:** Saturated fatty acids (SFA) profile (mean ± SD) in colostrum from Charolaise cows assigned to control (n = 10) and supplementation (n = 10; diet supplemented with soy lecithin) group.

Fatty Acids (g/100 g)	Group		*p*-Value
	Control	Supplementation	
	X ± SD	X ± SD	
C4:0	0.27 ± 0.14	0.45 ± 0.25	0.079
C6:0	0.35 ± 0.14	0.56 ± 0.15	0.005
C8:0	0.35 ± 0.06	0.44 ± 0.07	0.010
C10:0	1.24 ± 0.17	1.33 ± 0.10	0.178
C12:0	2.59 ± 0.34	2.54 ± 0.32	0.750
C14:0	14.91 ± 1.28	14.38 ± 1.69	0.460
C15:0	0.79 ± 0.07	1.03 ± 0.31	0.033
C16:0	49.75 ± 3.51	47.07 ± 5.47	0.373
C17:0	0.88 ± 0.23	0.94 ± 0.35	0.680
C18:0	4.15 ± 0.99	5.97 ± 1.89	0.021
Total SFA	75.28 ± 2.88	74.71 ± 5.05	0.949

X: means; ±SD: standard deviation; SFA: Saturated Fatty Acids.

**Table 4:** Unsaturated fatty acids (UFA) profile (mean ± SD) in colostrum from Charolaise cows assigned to control (n = 10) and supplementation (n = 10; diet supplemented with soy lecithin) group.

Fatty Acids (g/100 g)	Group		*p*-Value
	Control	Supplementation	
	X ± SD	X ± SD	
C14:1	1.33 ± 0.39	1.29 ± 0.19	0.825
C15:1	0.17 ± 0.04	0.20 ± 0.09	0.318
C16:1	3.81 ± 0.39	2.02 ± 0.61	0.005
C17:1	0.53 ± 0.09	0.48 ± 0.07	0.139
C18:1 n-9c	15.58 ± 2.79	17.51 ± 3.99	0.251
C18:1 n-9t	ND	0.48 ± 0.09	0.000
C18:1 n-11t	0.33 ± 0.08	ND	0.000
C18:2 n-6	1.35 ± 0.16	1.59 ± 0.30	0.049
*c*-9, *t*-11 (CLA)	0.26 ± 0.08	0.35 ± 0.14	0.103
C18:3 n-3	0.54 ± 0.09	0.65 ± 0.29	0.270
C20:4 n-6	0.28 ± 0.11	0.25 ± 0.07	0.465
C20:5 n-3 (EPA)	0.21 ± 0.07	0.18 ± 0.07	0.519
Remaining acids	0.33 ± 0.17	0.29 ± 0.11	0.128
Total UFA	24.72 ± 2.57	25.29 ± 4.26	0.342

ND—not detected. X: means; ±SD: standard deviation; CLA: Conjugated Linoleic Acid; EPA: Eicosapentaenoic Acid; UFA: Unsaturated Fatty Acids.

**Table 5:** IgG concentration (mean ± SD) in calves’ serum, mothered by control (n = 10) and supplementation (n = 10; diet supplemented with soy lecithin) Charolaise cows, on day 3rd, 7th, 14th, and 21st after birth.

Days of Life	IgG Concentration (g/L)		*p*-Value
	Control	Supplementation	
	X ± SD	X ± SD	
3rd	20.47 ± 10.28	21.48 ± 5.32	0.797
7th	18.42 ± 8.98	20.39 ± 5.07	0.575
14th	12.33 ± 5.52	18.20 ± 4.83	0.029
21st	15.03 ± 6.20	17.40 ± 3.98	0.350

X: means; ±SD: standard deviation.

**Table 6:** Serum total protein concentration (mean ± SD) in calves’ serum, mothered by control (n = 10) and supplementation (n = 10; diet supplemented with soy lecithin) Charolaise cows, on day 3rd, 7th, 14th, and 21st after birth.

Days of Life	STP concentration (g/L)		*p*-Value
	Control	Supplementation	
	X ± SD	X ± SD	
3rd	66.66 ± 10.61	67.39 ± 12.88	0.897
7th	58.92 ± 7.47	61.12 ± 11.36	0.634
14th	58.91 ± 7.13	57.73 ± 8.95	0.762
21st	57.31 ± 9.96	59.06 ± 11.78	0.739

X: means; ±SD: standard deviation.
